# Computational Psychometrics for Modeling System Dynamics during Stressful Disasters

**DOI:** 10.3389/fpsyg.2017.01401

**Published:** 2017-08-15

**Authors:** Pietro Cipresso, Alessandro Bessi, Desirée Colombo, Elisa Pedroli, Giuseppe Riva

**Affiliations:** ^1^Applied Technology for Neuro-Psychology Laboratory, Istituto Auxologico Italiano Milan, Italy; ^2^Department of Psychology, Catholic University of the Sacred Heart Milan, Italy; ^3^Information Sciences Institute, University of Southern California, Marina del Rey CA, United States

**Keywords:** modeling, psychological stress, computational psychometrics, communication, psychometrics, disaster management

## Abstract

Disasters can be very stressful events. However, computational models of stress require data that might be very difficult to collect during disasters. Moreover, personal experiences are not repeatable, so it is not possible to collect bottom-up information when building a coherent model. To overcome these problems, we propose the use of computational models and virtual reality integration to recreate disaster situations, while examining possible dynamics in order to understand human behavior and relative consequences. By providing realistic parameters associated with disaster situations, computational scientists can work more closely with emergency responders to improve the quality of interventions in the future.

## Introduction

Communication models could be one of the main resources to forecast scenarios and to have action plans ready for better interventions and effective policies based on realistic human behavior (Clark et al., 2001; Peterson et al., 2003; Cipresso et al., 2015b). As a matter of fact, information exchange between the various emergency operators plays a fundamental role, and it assumes further importance when international humanitarian relieves are needed (McCall and Salama, 1999; Connorton et al., 2012).

The classic Shannon and Weaver communication model includes a transmitter, a receiver, and feedback. More in general this a transmission model consisting of five elements: an information source, which produces a message; a transmitter, which encodes the message into signals; a channel, to which signals are adapted for transmission; a receiver, which decodes (reconstructs) the message from the signal; a destination, where the message arrives. In later formulations, more sophisticated elements and analyses were taken into account, consisting predominantly of mutual information processes (McQuail and Windahl, 2015; Shannon and Weaver, 2015). More recently, networked models of communication are attempting to provide further analyses to the theoretical modeling and application. Problems with empirical models mainly arise from the fact that, even if related to communication processes during the events, they are measured *a priori* or *a posteriori*, but never during the actual events. Some recent studies used social media crawlers, in particular Twitter data, to understand how communication works during disasters (Houston et al., 2015; Jung et al., 2015; Parsons et al., 2015; Takahashi et al., 2015). However, the psychological elements affecting individual behavior during a disaster have yet to be studied comprehensively. Moreover, a link between individual and social levels is still missing, and, in our opinion, a better understanding of this link is needed.

In the following paragraphs, we aim at proposing an innovative class of methods to help experts in the study of communication processes, forecasting, and intervention during a disaster. Contrary to the collective imagination, people rarely panic in the aftermath of a disaster: Rather than acting as individuals, the crowd behavior is influenced by the pre-existing social bonds and norms, leading to the development of a sense of “we-ness” linked to the perception of a shared fate (Johnson et al., 1994; Aguirre et al., 1998; Perry et al., 2001; Clarke, 2002; Drury et al., 2006). The exposure to natural disasters may instead lead to the development of physical and emotional trauma, such as depression, post-traumatic stress disorder (PTSD), anxiety disorders or psychological stress (Tang et al., 2014; Parker et al., 2016). According to our intent, we will specifically focus on psychological stress as a possible consequence of the occurrence of natural disasters.

## Complex System And The Analyses Of Stress As Emergent Phenomena

As stated by Levine (2005), the main problem with the definition of stress is that stress is a complex and multidimensional concept. As a matter of fact, all the existing definitions focus only on some components: The input, namely, the stressor; the processing system, including the individual experience and interpretation of the stressor, and the output (Levine and Ursin, 1991).

Stressors are events that are perceived as threats for the psychological and physiological integrity of a person, resulting in physiological, behavioral and/or cognitive responses which aim to reinstate the ideal homeostatic balance (McEwen, 2000). According to Cohen et al. (2007) psychological stress occurs when an individual perceives that the environmental demands exceed her or his adaptive ability to deal with them. Effective coping strategies are essential in order to rapidly and efficiently respond to such events. If the responses to the stressors are inadequate, the biological costs may become too high (de Kloet et al., 2005) and negative emotional responses, such as feelings of anxiety, may be elicited (Cohen et al., 2007).

An important phenomenon to be evaluated, while considering stress and natural disasters, is stress contagion, which can be studied in the literature that deals with emotions and their transmission: Human emotions are indeed significantly affected by social contacts (Hill et al., 2010) and this contagion has been explained in terms of social sensitivity (Guastello et al., 2006). For instance, the interaction with a subject experiencing acute stress can consequently cause some physiological changes in the observer (Butler et al., 2003; Buchanan et al., 2012).

In the field of disaster communication, social network analysis are often used to understand the dynamics of such communication and they are based on the analyses of data or big data (Hossain and Feng, 2016; Kano et al., 2016). Differently, another way to extensively investigate stress contagion is to use the same concept of networks with social simulation (Guo and Kapucu, 2015). The use of agent-based models makes it possible to give “intelligence” to the network nodes, allowing the emergence of big properties based on simple rules (Serrano and Iglesias, 2016). The surprising, yet confirmed, hypotheses that result, might advance our understanding of the relationship between human behavior and social emergence.

During a natural disaster, communications among the responders and among the population affect people’s behaviors (actions), emotions (internal aspects), the community at large (social aspects), the environment (physical constraints), and the generally accepted and used set of rules (properties within which people move and act in their physical and cognitive domains). Several models of human behavior and of communications during a disaster have been developed, but, of course, no models based completely on data from the field are available. The possible solution would be to replicate a disaster situation in a laboratory, where it would be possible to record behavioral, emotional, physiological, verbal, and other data during a specific situation. Importantly, clinicians face the same problem when dealing with PTSD patients (Fernandez et al., 2016).

Consistently with these observations, virtual reality (VR) may constitute a valid tool to bridge this gap, as it has already been used in several applications and it has been validated in clinical and other settings (Botella et al., 2015; Rizzo et al., 2015). Numerous studies have demonstrated that VR has effects on the psychophysiological system. For instance, physiological responses were found in computer-generated stress tasks (Kotlyar et al., 2008) as well as in exposure treatment programs for acrophobia (fear of heights) and PTSD (Difede and Hoffman, 2002; Delahaye et al., 2007; Gerardi et al., 2010; Meyerbröker and Emmelkamp, 2010; North and North, 2016). VR has a success rate equal to that of *in vivo* exposure, and it has been also demonstrated that, during VR exposure, the somatic correlates (heart rate, respiratory rate) of an anxiety response (or panic-like response) are elicited. Three well-known meta-analyses (Parsons and Rizzo, 2008; Powers et al., 2008; Opriş et al., 2012) also demonstrated the efficacy of VR in the treatment of stress-related disorders, specifically PTSD.

In virtual reality exposure (VRE), users are immersed within a computer-generated simulation or in virtual environments (VE) that updates in a natural way based on the motions of the user’s head and/or body. When users are immersed in a VE, they can be systematically exposed to specific, feared stimuli within a contextually relevant setting (Rothbaum et al., 2001). VRE fits well with the emotional processing model, which posits that the fear of networking must be activated through confrontation with threatening stimuli and that new, incompatible, disconfirming information must be added into the emotional network (Wilhelm et al., 2005).

Recently, however, Cipresso (2015) suggested that VR could be more than a tool to provide exposure and desensitization: VR could be further used to create a model of behavior dynamics, allowing to take into account the spread of behaviors in specific situations.

There have been very few efforts to address the problem of how social and physical interactions contribute to the engagement of relations between the world and oneself. Previous studies have undertaken behavior and emotive emergence by coupling human-centered perception with interacting human physiological and behavioral signals (Cipresso, 2015). The first issue to consider when investigating embodied interaction between humans and systems is the social and physical interface with the human sensory system, considering both innovative devices and communications paradigms. Wearable biomedical sensors and systems consisting of unobtrusive electronic sensing and/or textile-based interfaces is a novel artificial embodiment concept in which both vital and/or behavioral signs and relevant information from the environment can be determined (Mauri et al., 2010; Cipresso et al., 2014a).

In the last few decades, the costs associated with a VR system and creating VE have decreased significantly. Currently, there are several software options that can be used if one only has moderate computational skills. Moreover, VR software can be integrated with biosensors for psychophysiological recording, motion detection systems for recording behavioral movements, and other devices for speech, eye movements, body detection and gesture recognition, affective states, and many others (Riva et al., 2011; Cipresso et al., 2014b, 2016).

With VR, we can replicate a disaster situation in a laboratory setting while recording internal and behavioral states in the participants. The understanding of participants in disaster situations could provide the necessary information for a computational model that considers all of the elements that might affect communications in that specific situation. In particular, since disasters represent traumatic situations for those who are experiencing them, the possible collection of emotional data to be included in a model is a crucial aspect that should be considered.

## Understanding From The Bottom-Up: Emulation And Simulation

Since VR can be used to understand the basic rules of behavior during an emulated disaster, we can build a model that can integrate artificial agents, process the interactions among them, and indicate the different ways of interaction that affect the system’s equilibrium (Cipresso, 2015; Cipresso et al., 2015a). However, herein, simulations study the communication dynamics during disasters rather than giving indications of how to behave during the event. VR would make it possible including several parameters and collecting the main aspects of a behavior during a disaster in a computational model of communication. A computational, communication agent-based model is composed of an artificial environment, agents, and rules that represent the real situation to be simulated (Gilbert and Terna, 2000; Smith and Conrey, 2007; Gilbert, 2008). This is not realistic for a simulation model, but it is parameterized to be responsive with respect to crucial parameters, such as emotional variations, crowding behavior, and other parameters that can be considered (Smith and Conrey, 2007). The use of VR allows the *a priori* setting of different kinds of parameters on the basis of experiments performed on several participants. Practically, the researcher emulates a specific situation several times, such as a fire, and sets the computational model on the basis of the emotional and behavioral parameters observed in the participants. To set these parameters, it is important to refer to specific literature references. For example, we know that people experiencing stress tend to diffuse the stress, so we will determine when each event stresses most of the participants much more than usual, and we will rank all of the events on the basis of the stress levels in the participants. By conducting this simple ranking, we can determine which events during the disaster are more prone to disseminate stress communication, and we can use this information *a priori* to manage the communication in a better way. An example has been the recent diffusion of stress communication and behavior among a huge group of fans watching the Champions League final match of Juventus in a square of Turin, in the north of Italy. During the second half of the competition, the crowd was suddenly interrupted by a loud noise, whose reason has not been clearly identified, probably a huge firecracker. Listening to this sort of bang, people started running and screaming thinking to a terrorist attack. According to the authorities, the crowd was taken by panic, fearing that the loud noise was caused by attackers. The situation brought to diffuse stress, and inopportune behaviors caused different accidents in the area: More than 1,500 were injured and reported cuts and light contusions, needing hospital treatments. The detailed news can be read in international newspapers, such as “The Guardian”^1^ and “The Telegraph”^2^, under the title “More than 1,500 Juventus fans injured in stampede in Turin.”

A more sophisticated approach that includes several parameters in the computational model is to run simulations with multiple participants and to integrate real data from simulated evacuations with the simulated data, in a way that allows the computational model to fix also the unknown variables that arise only in the real settings. The availability of the VR platform for multi-user cooperation allows the concurring emulation of a complex scenario that provides an empowered way to collect data for the specific situation being studied. In addition, the use of sensors, such as electrocardiographic or galvanic skin response sensors, during the VR emulation can further improve the quality of the data collected for the model parameterization. In particular, biosensors can provide precise and important information related to emotional cues during the events, and this information could be important in forecasting panic, which is a principal component of a computational model.

## Systemic Emergence: A Simulation Example With System Dynamics Modeling

**FIGURE 1:**
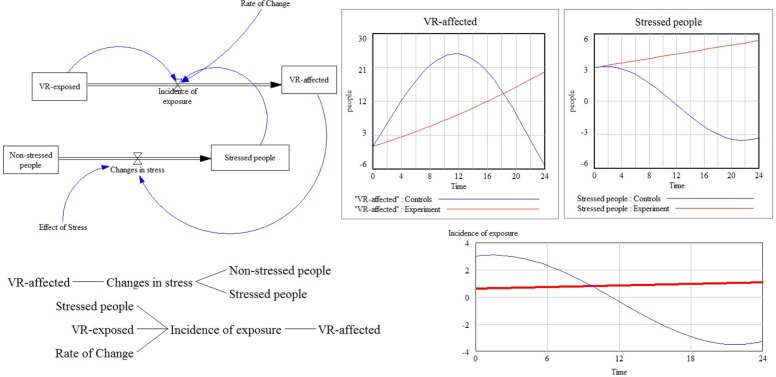
An artificial simulation with system dynamics models. The figure represents an example of modeling simulation by using different kind of parameter from a virtual environment to a computational model. VR exposure produce an effect only in the experimental group confirming that to expose people to VR stressors has an effect only when a combination of personal stress and virtual exposure is combined.

System dynamics simulation is useful in developing a deeper understanding of the non-linear behavior of complex systems, such as the stress dynamics during disasters. The model acts over simulated time by means of internal feedback loops and time delays that are mediated by the adoption of the classic “stocks” and “flows” used in system dynamics models. **Figure 1** shows an example of a simulation, and the model that contains the VR-exposed people and non-stressed people exposed to a stressor is highlighted on the left side of the figure. Also, we defined an *incidence of exposure* based on arbitrary initial conditions as (Rate of Change × Stressed people × VR-exposed/1000) and *changes in stress* as (0.1+Effect of Stress × VR-affected). These parameters also can be changed by means of specific experiments in VE.

The simulation results on the right side of the figure show that both exposure and stress must be considered together to comply with the huge complexity in this non-linear system. These models provide a straightforward way to address the practical problem of defining system dynamics in complex systems. Their use with stressful disasters would be very difficult without the identification of real parameters from the field. VR can provide an effective way to estimate several behavioral parameters in stressful situations, representing therefore an interesting perspective and a future challenge to be tested and considered in the field.

## Conclusion

By using VR, we provided the concept of building a computational model of stress based on agent-based, artificial simulation and realistic information collected through emulated simulation. A model always represents a small part of reality and it is, by definition, subject to errors and recurrent adjustments. However, we provided a simple and effective way to overcome some limitations intrinsic in the modeling. In fact, VR constitutes a valid alternative to emulate simulation in a safe and controlled way within a laboratory setting. In addition, VR allows the use of integrated sensors for the collection of physiological and behavioral parameters, improving the integration of data into the computational model. Even if there are some limitations of the computational modeling, we believe that the concept of having a practical instrument to prepare stressful scenarios for several kinds of events may be very useful. In particular, this approach could have an important role in integrating current empirical models based on real data collected *a posteriori* in the hope of preventing future errors and of improving the intervention with a computational tool.

## Author Contributions

PC wrote the first draft of the article. PC and AB worked on the computational model. DC and EP revised the article and addressed reviewers’ comments including new parts and idea into the article. PC and GR conceived the idea. All the authors revised the article and approved the final version.

## Conflict of Interest Statement

The authors declare that the research was conducted in the absence of any commercial or financial relationships that could be construed as a potential conflict of interest.

## Footnotes

1 https://www.theguardian.com/football/2017/jun/03/juventus-fans-injured-turin-square-panic-firecrackers

2 http://www.telegraph.co.uk/news/2017/06/04/hundreds-juventus-fans-injured-stampede-turin/

## References

[B1] Aguirre B. E. Wenger D. Vigo G. (1998). A test of the emergent norm theory of collective behavior. *Sociol. Forum* 13 301–320. 10.1023/A:1022145900928

[B2] Botella C. Serrano B. Baños R. M. Garcia-Palacios A. (2015). Virtual reality exposure-based therapy for the treatment of post-traumatic stress disorder: a review of its efficacy, the adequacy of the treatment protocol, and its acceptability. *Neuropsychiatr. Dis. Treat.* 11 2533–2545. 10.2147/NDT.S89542 26491332 PMC4599639

[B3] Buchanan T. W. Bagley S. L. Stansfield R. B. Preston S. D. (2012). The empathic, physiological resonance of stress. *Soc. Neurosci.* 7 191–201. 10.1080/17470919.2011.588723 21777106

[B4] Butler E. A. Egloff B. Wilhelm F. H. Smith N. C. Erickson E. A. Gross J. J. (2003). The social consequences of expressive suppression. *Emotion* 3 48–67. 10.1037/1528-3542.3.1.48 12899316

[B5] Cipresso P. (2015). Modeling behavior dynamics using computational psychometrics within virtual worlds. *Front. Psychol.* 6:1725 10.3389/fpsyg.2015.01725 PMC4635205 26594193

[B6] Cipresso P. Matic A. Giakoumis D. Ostrovsky Y. (2015a). Advances in computational psychometrics. *Comput. Math. Methods Med.* 2015:418683 10.1155/2015/418683 PMC4539436 26346251

[B7] Cipresso P. Matic A. Lopez G. (2014a). “Pervasive computing paradigms for mental health,” in *Proceedings of the 4th International Symposium MindCare 2014* (Tokyo: Springer International Publishing). 10.1007/978-3-319-11564-1

[B8] Cipresso P. Serino S. Giglioli I. A. C. Giuliano I. Borra D. Farina A. (2014b). *Low-Cost Motion-Tracking for Computational Psychometrics Based on Virtual Reality.* New York, NY: Springer 137–148. 10.1007/978-3-319-13969-2_11

[B9] Cipresso P. Serino S. Riva G. (2016). Psychometric assessment and behavioral experiments using a free virtual reality platform and computational science. *BMC Med. Inform. Decis. Mak.* 16:37 10.1186/s12911-016-0276-5 PMC4799532 26992890

[B10] Cipresso P. Villani D. Repetto C. Bosone L. Balgera A. Mauri M. (2015b). Computational psychometrics in communication and implications in decision making. *Comput. Math. Methods Med.* 2015:985032 10.1155/2015/985032 PMC4538966 26339285

[B11] Clark J. S. Carpenter S. R. Barber M. Collins S. Dobson A. Foley J. A. (2001). Ecological forecasts: an emerging imperative. *Science* 293 657–660. 10.1126/science.293.5530.657 11474103

[B12] Clarke L. (2002). Panic: myth or reality? *Contexts* 1 21–27. 10.1525/ctx.2002.1.3.21

[B13] Cohen S. Janicki-Deverts D. Miller G. E. (2007). Psychological stress and disease. *JAMA* 298 1685–1687. 10.1001/jama.298.14.1685 17925521

[B14] Connorton E. Perry M. J. Hemenway D. Miller M. (2012). Humanitarian relief workers and trauma-related mental illness. *Epidemiol. Rev.* 34 145–155. 10.1093/epirev/mxr026 22180469

[B15] de Kloet E. R. Joels M. Holsboer F. (2005). Stress and the brain: from adaptation to disease. *Nat. Rev. Neurosci.* 6 463–475. 10.1038/nrn1683 15891777

[B16] Delahaye M. Mager R. Stefani O. Bekiaris E. Studhalter M. Traber M. Bullinger A. H. (2007). AKROPHOBIA treatment using virtual environments: evaluation using real-time physiology. *Paper Presented at the International Conference on Universal Access in Human-Computer Interaction* Berlin.

[B17] Difede J. Hoffman H. G. (2002). Virtual reality exposure therapy for World Trade Center post-traumatic stress disorder: a case report. *Cyberpsychol. Behav.* 5 529–535. 10.1089/109493102321018169 12556115

[B18] Drury J. Cocking C. Reicher S. (2006). Every man for himself-or for the group? How crowd solidarity can arise in an emergency: an interview study of disaster survivors. *Paper Presented at the Group and Intergroup Relations Pre-Conference, Society for Personality and Social Psychology 7th Annual Meeting* Palm Springs, CA.

[B19] Fernandez C. A. Vicente B. Marshall B. D. Koenen K. C. Arheart K. L. Kohn R. (2016). Longitudinal course of disaster-related PTSD among a prospective sample of adult Chilean natural disaster survivors. *Int. J. Epidemiol.* 46 440–452. 10.1093/ije/dyw094 PMC5837490 27283159

[B20] Gerardi M. Cukor J. Difede J. Rizzo A. Rothbaum B. O. (2010). Virtual reality exposure therapy for post-traumatic stress disorder and other anxiety disorders. *Curr. Psychiatry Rep.* 12 298–305. 10.1007/s11920-010-0128-4 20535592

[B21] Gilbert G. N. (2008). *Agent-Based Models.* Thousand Oaks, CA: Sage Publications 10.4135/9781412983259

[B22] Gilbert N. Terna P. (2000). How to build and use agent-based models in social science. *Mind Soc.* 1 57–72. 10.1007/BF02512229

[B23] Guastello S. J. Pincus D. Gunderson P. R. (2006). Electrodermal arousal between participants in a conversation: nonlinear dynamics and linkage effects. *Nonlinear Dynamics Psychol. Life Sci.* 10 365–399. 16762177

[B24] Guo X. Kapucu N. (2015). Examining coordination in disaster response using simulation methods. *J. Homel. Sec. Emerg. Manag.* 12 891–914. 10.1515/jhsem-2014-0092

[B25] Hill A. L. Rand D. G. Nowak M. A. Christakis N. A. (2010). Emotions as infectious diseases in a large social network: the SISa model. *Proc. Biol. Sci.* 277 3827–3835. 10.1098/rspb.2010.1217 20610424 PMC2992714

[B26] Hossain L. Feng S. (2016). Disaster network science: research and applications. *Front. Commun.* 1:1 10.3389/fcomm.2016.00001

[B27] Houston J. B. Hawthorne J. Perreault M. F. Park E. H. Goldstein Hode M. Halliwell M. R. (2015). Social media and disasters: a functional framework for social media use in disaster planning, response, and research. *Disasters* 39 1–22. 10.1111/disa.12092 25243593

[B28] Johnson N. R. Feinberg W. E. Johnston D. M. (1994). “Microstructure and panic: the impact of social bonds on individual action in collective flight from the Beverly Hills Supper Club fire,” in *Disasters, Collective Behavior and Social Organizations* eds Dynes R. R. Tierney K. J. (Newark, NJ: University of Delaware Press) 168–189.

[B29] Jung C.-T. Tsou M.-H. Issa E. (2015). Developing a real-time situation awareness viewer for monitoring disaster impacts using location-based social media messages in Twitter. *Paper Presented at the International Conference on Location-Based Social Media Data* Athens, GA.

[B30] Kano M. Wood M. M. Siegel J. M. Bourque L. B. (2016). “Disaster research and epidemiology,” *Disaster Medicine: Comprehensive Principles and Practicesin* eds Koenig K. L. Schultz C. H. (Cambridge: Cambridge University Press). 10.1017/CBO9781139629317.004

[B31] Kotlyar M. Donahue C. Thuras P. Kushner M. G. O’Gorman N. Smith E. A. (2008). Physiological response to a speech stressor presented in a virtual reality environment. *Psychophysiology* 45 1034–1037. 10.1111/j.1469-8986.2008.00690.x 18778321

[B32] Levine S. (2005). Developmental determinants of sensitivity and resistance to stress. *Psychoneuroendocrinology* 30 939–946. 10.1016/j.psyneuen.2005.03.013 15958281

[B33] Levine S. Ursin H. (1991). *What is Stress? Stress—Neurobiology and Neuroendocrinology.* New York, NY: Marcel Dekker.

[B34] Mauri M. Magagnin V. Cipresso P. Mainardi L. Brown E. N. Cerutti S. (2010). Psychophysiological signals associated with affective states. *Paper Presented at the 2010 Annual International Conference of the IEEE Engineering in Medicine and Biology* New York, NY 10.1109/IEMBS.2010.5627465 PMC3059749 21096828

[B35] McCall M. Salama P. (1999). Selection, training, and support of relief workers: an occupational health issue. *Br. Med. J.* 318 113–116. 10.1136/bmj.318.7176.113 9880288 PMC1114577

[B36] McEwen B. S. (2000). *Stress, Definitions and Concepts of. Encyclopedia of Stress* Vol. 3 San Diego, CA: Academic Press 508–509

[B37] McQuail D. Windahl S. (2015). *Communication Models for the Study of Mass Communications.* Abingdon: Routledge.

[B38] Meyerbröker K. Emmelkamp P. M. (2010). Virtual reality exposure therapy in anxiety disorders: a systematic review of process-and-outcome studies. *Depress. Anxiety* 27 933–944. 10.1002/da.20734 20734361

[B39] North M. M. North S. M. (2016). “Virtual reality therapy,” in *Computer-Assisted and Web-Based Innovations in Psychology, Special Education, and Health* eds Luiselli J. K. Fischer A. J. (Elsevier: Amsterdam).

[B40] Opriş D. Pintea S. García-Palacios A. Botella C. Szamosközi Ş. David D. (2012). Virtual reality exposure therapy in anxiety disorders: a quantitative meta-analysis. *Depress. Anxiety* 29 85–93. 10.1002/da.20910 22065564

[B41] Parker G. Lie D. Siskind D. J. Martin-Khan M. Raphael B. Crompton D. (2016). Mental health implications for older adults after natural disasters–a systematic review and meta-analysis–CORRIGENDUM. *Int. Psychogeriatr.* 28 21 10.1017/S1041610215001465 26349523

[B42] Parsons S. Atkinson P. M. Simperl E. Weal M. (2015). “Thematically analysing social network content during disasters through the lens of the disaster management lifecycle,” in *Proceedings of the 24th International Conference on World Wide Web Conferences Steering Committee* (Florence: ACM) 18–22. 10.1145/2740908.2741721

[B43] Parsons T. D. Rizzo A. A. (2008). Affective outcomes of virtual reality exposure therapy for anxiety and specific phobias: a meta-analysis. *J. Behav. Ther. Exp. Psychiatry* 39 250–261. 10.1016/j.jbtep.2007.07.007 17720136

[B44] Perry R. W. Lindell M. K. Tierney K. J. (2001). *Facing the Unexpected: Disaster Preparedness and Response in the United States.* Washington, DC: Joseph Henry Press.

[B45] Peterson G. D. Cumming G. S. Carpenter S. R. (2003). Scenario planning: a tool for conservation in an uncertain world. *Conserv. Biol.* 17 358–366. 10.1046/j.1523-1739.2003.01491.x

[B46] Powers M. B. Vedel E. Emmelkamp P. M. (2008). Behavioral couples therapy (BCT) for alcohol and drug use disorders: a meta-analysis. *Clin. Psychol. Rev.* 28 952–962. 10.1016/j.cpr.2008.02.002 18374464

[B47] Riva G. Gaggioli A. Grassi A. Raspelli S. Cipresso P. Pallavicini F. (2011). NeuroVR 2-A free virtual reality platform for the assessment and treatment in behavioral health care. *Stud. Health Technol. Inform.* 163 493–495. 21335845

[B48] Rizzo A. Cukor J. Gerardi M. Alley S. Reist C. Roy M. (2015). Virtual reality exposure for PTSD due to military combat and terrorist attacks. *J. Contemp. Psychother.* 45 255–264. 10.1007/s10879-015-9306-3

[B49] Rothbaum B. O. Hodges L. F. Ready D. Alarcon R. D. (2001). Virtual reality exposure therapy for Vietnam veterans with posttraumatic stress disorder. *J. Clin. Psychiatry* 62 617–622. 10.4088/JCP.v62n0808 11561934

[B50] Serrano E. Iglesias C. A. (2016). Validating viral marketing strategies in Twitter via agent-based social simulation. *Expert Syst. Appl.* 50 140–150. 10.1016/j.eswa.2015.12.021

[B51] Shannon C. E. Weaver W. (2015). *The Mathematical Theory of Communication.* Champaign, IL: University of Illinois press.

[B52] Smith E. R. Conrey F. R. (2007). Agent-based modeling: a new approach for theory building in social psychology. *Pers. Soc. Psychol. Rev.* 11 87–104. 10.1177/1088868306294789 18453457

[B53] Takahashi B. Tandoc E. C. Carmichael C. (2015). Communicating on Twitter during a disaster: an analysis of tweets during Typhoon Haiyan in the Philippines. *Comput. Hum. Behav.* 50 392–398. 10.1016/j.chb.2015.04.020

[B54] Tang B. Liu X. Liu Y. Xue C. Zhang L. (2014). A meta-analysis of risk factors for depression in adults and children after natural disasters. *BMC Public Health* 14:623 10.1186/1471-2458-14-623 PMC4077641 24941890

[B55] Wilhelm F. H. Pfaltz M. C. Gross J. J. Mauss I. B. Kim S. I. Wiederhold B. K. (2005). Mechanisms of virtual reality exposure therapy: the role of the behavioral activation and behavioral inhibition systems. *Appl. Psychophysiol. Biofeedback* 30 271–284. 10.1007/s10484-005-6383-1 16167191

